# MoO_2.8_F_0.2_/MoO_2.4_F_0.6_ heterostructures for high-rate magnesium-ion battery cathodes

**DOI:** 10.1093/nsr/nwae390

**Published:** 2024-11-12

**Authors:** Ze He, Michael De Volder

**Affiliations:** Institute for Manufacturing, Department of Engineering, University of Cambridge, UK; Institute for Manufacturing, Department of Engineering, University of Cambridge, UK

Rechargeable magnesium batteries (RMBs) hold great promise for ‘beyond Li-ion’ batteries owing to their low cost, high volumetric capacity (3833 mA h cm^−3^) and the dendrite-free deposition behavior of the Mg anode. Nonetheless, the strong electrostatic interactions between the highly polarized magnesium ions and the host lattice result in sluggish electrochemical reaction kinetics [1]. Therefore, exploration for advanced cathode materials to achieve efficient Mg^2+^ storage is an important bottleneck. Molybdenum-based oxides are interesting RMB cathodes but their practical application is limited due to poor electrical conductivity and sluggish Mg^2+^ diffusivity. To date, significant efforts have been made to address these limitations by using a range of methods such as an increase in the interlayer spacing and pre-intercalation [2–4]. Although these strategies are encouraging, they have not achieved the performance enhancements that are needed to commercialize RMBs.

**Figure 1.**
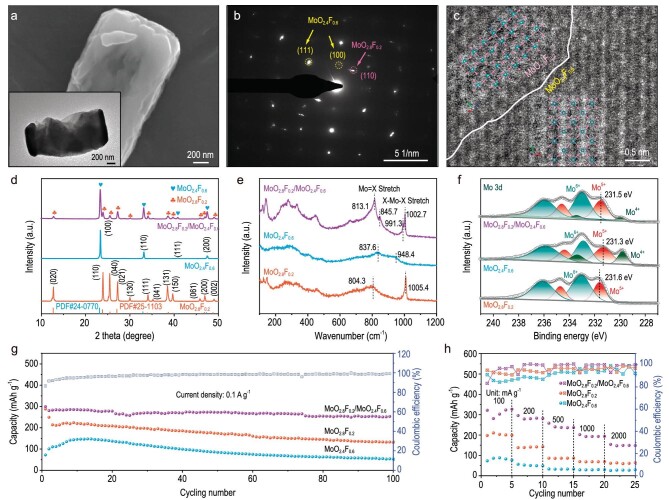
(a) Scanning electron microscopy image and transmission electron microscopy image, (b) selected area electron diffraction pattern and (c) high-angle annular dark-field scanning transmission electron microscopy image of o–c MoO_2.8_F_0.2_/MoO_2.4_F_0.6_ heterostructures. (d) X-ray diffraction patterns, (e) Raman spectra and (f) Mo 3d X-ray photoelectron spectroscopy spectra of o-MoO_2.8_F_0.2_, c-MoO_2.4_F_0.6_ and o–c MoO_2.4_F_0.6_/MoO_2.8_F_0.2_ heterostructures. (g) Cycling performances and (h) rate capabilities of the MoO_2.8_F_0.2_, MoO_2.4_F_0.6_ and MoO_2.8_F_0.2_/MoO_2.4_F_0.6_ electrodes. Reproduced with permission from [5].

In a recent study that was published in *National Science Review* [5], Prof. Liqiang Mai's group at Wuhan University of Technology modified MoO_6_ cathodes by replacing the oxygen partially with fluorine and they found that the difference in the electronegativity and valence electron configuration of fluorine led to a dual-phase orthorhombic phase MoO_2.8_F_0.2_ and cubic phase MoO_2.4_F_0.6_. This is evidenced in the paper by the use of electron microscopy and material characterization methods (see Fig. 1a–f) as well as simulations.

The authors propose that the orthorhombic MoO_2.8_F_0.2_ introduces molybdenum vacancies that unlock the inactive basal plane of the layered crystal structure while the cubic MoO_2.4_F_0.6_ increases the diffusion channel size, enhancing the ion diffusion coefficient in the material and resulting in improved rate performance. In addition, the dual-phase MoO_2.8_F_0.2_/MoO_2.4_F_0.6_ heterostructure increases the electron transport in the material, resulting in an overall improvement in the MoO_2.8_F_0.2_/MoO_2.4_F_0.6_ cathode rate performance (see Fig. 1h). These materials achieved 303.8 mAh g^−1^ at 0.1 A g^−1^ and maintained 154.1 mAh g^−1^ at 2 A g^−1^ (see Fig. 1h). Together with the very low capacity loss that was observed over 100 cycles (Fig. 1g) and the use of relatively abundant materials, this paper demonstrates an interesting path for the creation of new RMB cathodes. Further, their proposed methodology to design and characterize a dual-phase heterostructure offers a strategy for improving the kinetics of other battery systems.
